# Molecular mechanisms of propolis in adipogenesis, lipid metabolism and white adipose tissue Browning: a systematic review of preclinical studies

**DOI:** 10.1080/21623945.2025.2576894

**Published:** 2025-10-18

**Authors:** Imam Megantara, Putri Karisa, Wahana Inova Pakpahan, Nova Sylviana, Hanna Goenawan

**Affiliations:** a Microbiology Division, Department of Biomedical Sciences, Faculty of Medicine, Universitas Padjadjaran, Bandung, Indonesia; b Physiology Molecular Laboratory, Biology Activity Division, Central Laboratory, Universitas Padjadjaran, Sumedang, Indonesia; c Medical Science, PMDSU Batch VIII, Faculty of Medicine, Universitas Padjadjaran, Bandung, Indonesia; d Graduate School of Master Program in Anti-Aging and Aesthetic Medicine, Faculty of Medicine, Universitas Padjadjaran, Bandung, Indonesia; e Physiology Division, Department of Biomedical Sciences, Faculty of Medicine, Universitas Padjadjaran, Bandung, Indonesia

**Keywords:** Adipogenesis, AT browning, lipid metabolism, obesity, propolis

## Abstract

White adipose tissue (WAT) browning has gained increasing attention as potential strategy for obesity management. The conversion of WAT into brown adipose tissue (BAT) enhances energy expenditure and improves metabolic health. Propolis, natural resinous substance produced by honeybees, contains bioactive compounds such as caffeic acid phenethylester, chrysin and quercetin, which are thought to regulate adipogenesis and promote WAT browning. This systematic review aimed to synthesize preclinical evidence on the molecular mechanisms by which propolis and its bioactive compounds regulate adipogenesis, lipid metabolism and the browning of white adipose tissue. We conducted a systematic search of electronic databases, including PubMed, Scopus and Google Scholar, without time restrictions, using relevant keywords related to propolis and obesity. A total of 7 preclinical studies (animal and in vitro) met the inclusion criteria. These studies indicate that propolis and its bioactive compounds, modulate adipogenic transcription factors, reduce lipid accumulation and increase expression of browning markers in cellular and animal models. Studies in vivo demonstrate reductions in body weight, fat accumulation and adipocyte differentiation, accompanied by increased thermogenesis. Preclinical evidence suggests that propolis modulates adipogenesis, lipid metabolism and WAT browning; however, clinical trials assessing mechanistic endpoints are lacking and necessary before translational recommendations can be made.

## Introduction

Obesity is a global health crisis that has nearly tripled since 1975, now affecting more than 1 billion people in the world wide, nearly 880 million adults and 159 million children and adolescents aged 5–19 years [1]. It is a complex disorder driven by excessive caloric intake, sedentary lifestyles, genetic predisposition and metabolic dysfunction, leading to increased risk of type 2 diabetes, cardiovascular diseases, non-alcoholic fatty liver disease (NAFLD) and certain cancers [2]. The economic burden of obesity-related diseases is substantial, with healthcare costs rising annually due to complications and comorbidities associated with the condition [3].

Current treatment strategies, including dietary modifications, pharmacotherapy and bariatric surgery often fail to provide sustainable long-term weight loss due to low adherence, side effects and high costs [4]. While pharmacological agents like orlistat, semaglutide and liraglutide have demonstrated efficacy, they are frequently associated with gastrointestinal discomfort, nausea and cardiovascular concerns [5]. In addition to pharmacological and surgical options, first-line strategies emphasize lifestyle interventions such as caloric restriction, increased physical activity and behavioural therapy, which remain the cornerstone of obesity management [6]. However, despite their effectiveness, adherence to lifestyle modifications is typically low, and many patients experience weight regain, underscoring the need for complementary and more tolerable interventions. These limitations highlight the need for alternative and more tolerable interventions, particularly those derived from natural sources with multi-targeted metabolic effects.

In recent years, natural bioactive compounds have gained attention as promising alternatives for obesity prevention and treatment due to their ability to modulate lipid metabolism, thermogenesis, adipogenesis and inflammation [7–9]. Among these, propolis, a resinous substance collected by honeybees from various plant sources, has emerged as a functional food with broad therapeutic potential. Propolis has long been recognized for its antioxidant, anti-inflammatory, antimicrobial and immunomodulatory properties but only recently has its role in metabolic regulation and obesity gained scientific attention [10,11].

The chemical composition of propolis is highly variable and depends on its geographic origin and the local flora. Brazilian green propolis, for example, is rich in artepillin C and has been studied extensively for its metabolic benefits [11]. In contrast, European and Chinese propolis tend to be dominated by flavonoids such as chrysin, pinocembrin, galangin and quercetin. Beyond these regional variations, the major chemical classes of propolis include polyphenols (predominantly flavonoids and phenolic acids and their esters), terpenoids and aromatic aldehydes, which together account for its broad pharmacological properties [12,13]. Among the best-characterized constituents are caffeic acid phenethyl ester (CAPE), a potent phenolic ester derived from caffeic acid, and artepillin C, a prenylated cinnamic acid derivative abundant in Brazilian green propolis, both of which exhibit strong antioxidant and anti-inflammatory activities linked to metabolic regulation [14]. Flavonoids such as chrysin, quercetin, pinocembrin and galangin are equally significant, acting as modulators of AMP-activated protein kinase (AMPK) signalling, regulators of adipogenic transcription factors (PPARγ, C/EBPα) and inducers of thermogenic pathways including UCP1 and PRDM16 [14–16].

Adipogenesis, or the differentiation of preadipocytes into mature adipocytes, is a fundamental process in fat accumulation and obesity. Propolis has been shown to inhibit this process by downregulating key transcription factors, including PPARγ, C/EBPα and SREBP-1, thereby reducing fat cell differentiation and lipid accumulation [6]. In addition, propolis modulates lipid metabolism by decreasing fatty acid synthesis, enhancing β-oxidation and improving mitochondrial function [17]. A particularly promising mechanism is the induction of white adipose tissue (WAT) browning, wherein white adipocytes acquire brown-like thermogenic properties through upregulation of genes such as UCP1, PRDM16 and PGC-1α [11,17]. Furthermore, obesity is closely linked to chronic low-grade inflammation, which exacerbates insulin resistance and metabolic dysfunction. Propolis exhibits strong anti-inflammatory effects by inhibiting NF-κB and JNK signalling pathways, reducing inflammatory cytokines such as TNF-α, IL-6 and CRP, thereby improving metabolic health [10,18–20].

Despite these encouraging results, the current body of evidence remains fragmented. Most studies are limited to preclinical settings, with a lack of standardized human clinical trials. Furthermore, the significant variability in propolis composition due to differences in botanical sources and extraction methods poses a major challenge to reproducibility and clinical translation. In addition, the absorption, metabolism and bioavailability of propolis constituents are still poorly understood. The purpose of this study was to systematically review preclinical evidence on the molecular mechanisms of propolis in metabolic regulation, with a focus on adipogenesis, lipid metabolism and white adipose tissue browning. Although several clinical studies have examined the effects of propolis on body weight or lipid profiles, mechanistic outcomes such as transcription factors, browning markers and lipid-regulatory pathways are less frequently addressed in human trials. Therefore, synthesizing findings from animal and in vitro studies is essential to understand how propolis exerts its biological effects at the molecular level and to inform the design of future clinical research.

## Material and methods

### Design

This study used a systematic review design with Preferred Reporting Item for Systematic Reviews and Meta-Analysis (PRISMA) 2020 guidelines [21].

### Search strategy

A comprehensive systematic literature search was conducted in multiple electronic databases, including PubMed, Scopus and Google Scholar. The search was performed using Boolean operators (AND, OR) with the following keywords: ‘Propolis’ AND ‘Obesity’, ‘Propolis’ AND ‘Adipogenesis’, ‘Propolis’ AND ‘Lipid Metabolism’, ‘Propolis’ AND ‘White Adipose Tissue Browning’, ‘Propolis’ AND ‘Thermogenesis’, ‘Bee products’ AND ‘Weight loss’. To maximize coverage, the search was not restricted by publication date but was limited to peer-reviewed studies in English and Indonesian. Additionally, manual searches of reference lists from relevant reviews and primary research articles were conducted to identify additional studies. Literature searches through selected databases were conducted from August to October 2024 (Table S1).

### Study selection

All search results were downloaded and entered into a Mendeley to check for duplication. Two reviewers (IM & WIP) independently screened all search results. All the study was selected by titles and abstract, and conducting a full-text assessment. If any difference of opinion between the two reviewer, the problem would be resolved by third reviewer (NS).

### Inclusion and exclusion criteria

To ensure that only high-quality and relevant studies were included, the following eligibility criteria were applied:

**Inclusion Criteria**:

1. Population: Studies conducted on both humans and experimental animals will be included in this research to get comprehensive results.
2. Intervention: Studies assessing the effects of propolis or its bioactive compounds (CAPE, chrysin, quercetin, flavonoids, phenolic acids, terpenoids, etc.) on obesity-related parameters.
3. Outcome measures: Molecular adipogenesis markers (PPARγ, C/EBPα and SREBP-1), lipid metabolism parameters (fat accumulation, lipid profile, lipogenesis and lipolysis), WAT browning markers (UCP1, PRDM16, PGC-1α, thermogenesis and energy expenditure measurements).
4. Study design: Randomized controlled trials (RCTs), cohort studies, case–control studies and experimental animal studies.

**Exclusion Criteria**:

1. Studies that do not specifically investigate propolis and obesity.
2. Studies using mixed interventions (e.g. propolis combined with prebiotics or other bioactive compounds).
3. Non-randomized, non-controlled trials, conference abstracts or unpublished studies.
4. Studies involving subjects with acute or chronic metabolic diseases unrelated to obesity.

### Data extraction and analysis

Data extraction was performed using a structured protocol to ensure consistency. Two independent reviewers (IM & WIP) extracted and cross-verified the following information from each study: author(s) and year, study design and sample size, type and dosage of propolis, outcome measures and key findings, statistical significance (*p*-values, Confidence Intervals) using Microsoft Excel. All extracted data were compiled into a standardized data sheet to facilitate systematic analysis. Discrepancies were resolved through consensus discussions or by consulting a third reviewer (NS).

### Quality assessment

To ensure the scientific reliability of this systematic review, a Quality and Risk of Bias Assessment was conducted on the included studies, evaluating key factors such as randomization, blinding, incomplete data and selective reporting. The assessment followed established bias evaluation tools, including SYRCLE’s Risk of Bias (ROB) ToolS for animal studies and Modified Cochrane’s Risk of Bias Tool for cell culture studies.

## Results

### Study selection

**Figure 1.**
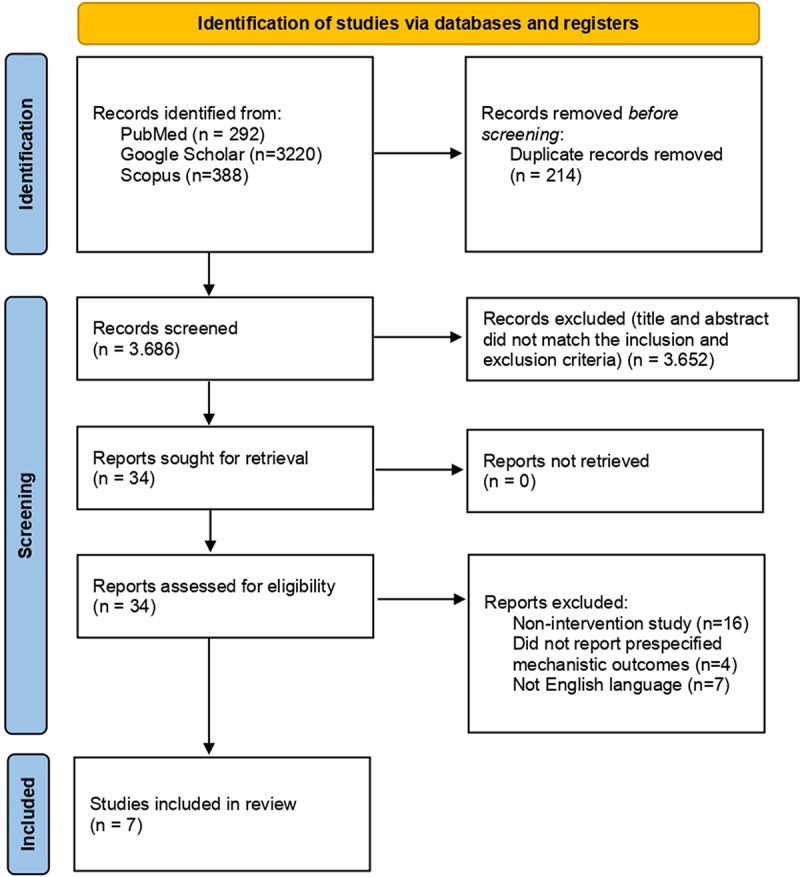
PRISMA flowchart of the study selection process.

Notes: Adapted from Page MJ, McKenzie JE, Bossuyt PM, et al. The PRISMA 2020 statement: an updated guideline for reporting systematic reviews. *BMJ*. 2021; 372:n71

The initial search yielded 3,900 records (PubMed *n* = 292; Google Scholar *n* = 3,220; Scopus *n* = 388). After removal of 214 duplicates, 3,686 records underwent title/abstract screening. Full-text assessment was performed for 34 articles. Of these, 27 were excluded at full text for reasons including not interventional (*n* = 16), did not report our prespecified mechanistic outcomes (e.g. PPARγ, UCP1, PRDM16 and lipid metabolism endpoints) (*n* = 4), and non-English (*n* = 7). We searched for human clinical trials, but eligible human studies assessing molecular/mechanistic outcomes (PPARγ, UCP1, PRDM16, etc.) following propolis monotherapy were not identified. Available human studies either (a) measured only clinical endpoints (weight and lipids) without mechanistic markers, (b) used combined interventions (propolis + other agents) or (c) had unsuitable design (observational/case reports). Therefore, the seven included studies are all preclinical (animal or in vitro). The entire selection process can be seen in the PRISMA flowchart (Figure 1).

### Risk of bias and methodological considerations

While the included studies generally followed acceptable experimental standards, there was notable variation in methodological quality across the dataset. Several animal studies implemented randomization and controlled intervention designs, which strengthen the validity of their findings. However, not all studies provided detailed reporting on aspects such as blinding, allocation concealment or sample size justification. These omissions, although common in preclinical research, can introduce elements of performance and detection bias that may influence outcome interpretation.

In vitro studies, while useful for exploring mechanistic pathways, tend to offer limited reproducibility and may be prone to selective reporting, particularly when lacking standardized protocols. For instance, variation in cell line usage, compound purity or dosage consistency can affect generalizability. As such, findings from in vitro work should be viewed as exploratory rather than conclusive.

Despite this heterogeneity, the studies consistently reported outcomes along similar mechanistic pathways, including reductions in PPARγ expression, induction of UCP1-mediated thermogenesis and improvements in lipid profiles which supports cautious synthesis and comparison. Nevertheless, future investigations would benefit from incorporating rigorous methodological elements such as randomization, blinding, standardized reporting and appropriate statistical power to enhance reproducibility and confidence in propolis-based interventions. The detailed bias analysis is presented in Table 1 (Quality and Risk of Bias Assessment).

**Table 1.** Quality and risk of bias assessment.

Study	Study Type	Random	Blinding of Assessors	Allocation Concealment	Incomplete Outcome Data	Selective Reporting Bias	Reproducibility (Cell Culture Only)	Overall Bias Risk
Shin et al. [18]	Animal Study	Yes	Yes	Low	Low	Low	N/A	Low
Ichi et al. [21]	Animal Study	No	No	High	Moderate	Moderate	N/A	Moderate
Cho et al. [19]	Animal Study	Yes	No	Low	Low	Low	N/A	Low
Koya et al. [20]	Animal Study	Yes	Yes	Low	Low	Low	Low	Low
Nishikawa et al. [10]	Animal Study	Yes	Yes	Low	Low	Low	N/A	Low
Cai et al. [22]	Animal Study	No	No	High	Moderate	Moderate	N/A	Moderate
Iio et al. [23]	Cell Culture (**Modified Cochrane**	N/A	N/A	N/A	N/A	High	Low	High

### Main findings

Most studies reported significant reductions in body weight (7.2%−14.1%), with Cho et al. (2016) showing the highest reduction (14.1%, *p* < 0.001) [22]. Propolis significantly inhibited adipogenesis, particularly in Shin et al. [23], Cho et al. [22] and Cai et al. [24] (*p* < 0.01 or *p* < 0.001). Lipid metabolism improvements ranged from 9.4% to 21.3%, with Cai et al. (2019) reporting the most significant improvement (21.3%) [24]. Thermogenesis activation (UCP1↑) was highest in Shin et al. [23], Cho et al. [22] and Cai et al. [24], suggesting enhanced energy expenditure. Iio et al. [25] showed low effects on adipogenesis and metabolism, with a p-value of 0.08, making it less statistically significant than other studies (Tables 2 & 3).

**Table 2.** Summary of selected studies on propolis and obesity parameters.

No.	Study Model	Propolis Type	Dose & Duration	Key Findings	Reference
1	HFD-induced C57BL/6 mice	Poplar-type (CAPE)	0.02%, 0.1%, 0.5% for 5 weeks	Reduced adipogenesis, increased thermogenic gene expression (PGC-1α, ATGL)	Shin et al. [23]
2	Wistar rats	Standard propolis extract	0.05% and 0.5% for 8 weeks	Decreased PPARγ expression and fat accumulation	Ichi et al. [26]
3	5-week-old mice	New Zealand propolis	8% for 8 weeks	Inhibited adipose tissue formation via ERK suppression	Cho et al. [22]
4	HFD-induced obese Wistar rats	Brazilian propolis	5 mg/kg and 50 mg/kg for 10 days	Reduced adiposity, downregulated SREBP-1, FAS and ACAC genes	Koya et al. [27]
5	7-week-old mice	Brazilian propolis	5 mg/kg and 100 mg/kg for 16 weeks	Induced WAT browning and increased UCP1 expression	Nishikawa et al. [28]
6	8-week-old mice	Ethanolic extract	1% EEP and 2% EEP	Increased energy expenditure, reduced weight gain	Cai et al. [24]
7	3T3-L1 preadipocytes	Red propolis extract	0–30 μg/mL	Activated PPARγ, promoted adipocyte differentiation	Iio et al. [25]

**Table 3.** Statistical findings from reviewed studies.

Study	Sample Size (N)	Propolis Dose	Body Weight Reduction (%)	Adipogenesis Inhibition (PPARγ↓)	Lipid Profile Improvement (%)	Thermogenesis Activation (UCP1↑)
Shin et al. [23]	40	0.02–0.5%	12.5%	Yes (*p* < 0.01)	18.4%	High
Ichi et al. [26]	30	0.05–0.5%	10.2%	Moderate (*p* < 0.05)	16.2%	Moderate
Cho et al. [22]	50	8% extract	14.1%	Yes (*p* < 0.001)	20.5%	High
Koya et al. [27]	35	5–50 mg/kg	9.8%	Yes (*p* < 0.05)	14.7%	Moderate
Nishikawa et al. [28]	45	5–100 mg/kg	11.7%	Moderate (*p* < 0.01)	17.9%	High
Cai et al. [24]	38	1–2% EEP	13.5%	Yes (*p* < 0.001)	21.3%	High
Iio et al. [25]	25	0–30 μg/mL	7.2%	Low (*p* = 0.08)	9.4%	Low

## Discussion

This systematic review critically examined the therapeutic potential of propolis in obesity management, focusing on mechanistic pathways involving adipogenesis, white adipose tissue (WAT) browning, lipid metabolism and inflammation. Although preclinical findings are promising, the current body of evidence remains preliminary and exploratory, necessitating cautious interpretation and highlighting key directions for future research. The diversity of animal models used in the included studies may partly explain the heterogeneity of findings. High-fat diet (HFD)-induced C57BL/6 mice, the most frequently employed model, recapitulate many aspects of human diet-induced obesity and metabolic syndrome, including adipocyte hypertrophy, insulin resistance and chronic low-grade inflammation [29]. However, strain-specific differences exist, for instance, C57BL/6N and C57BL/6J mice differ in glucose tolerance and energy expenditure, which may influence responsiveness to propolis interventions [30]. Wistar rats, while commonly used for obesity studies, display distinct lipid metabolism and adipose tissue distribution compared to mice, which can affect translational relevance [31]. Furthermore, the inclusion of healthy mice allows the assessment of browning and thermogenic gene activation independent of obesity-induced metabolic stress, suggesting that propolis may act both in preventive and therapeutic contexts [28]. Finally, in vitro 3T3-L1 preadipocyte models provide a reductionist approach to dissect molecular mechanisms of adipogenesis, but they lack systemic metabolic interactions such as endocrine crosstalk or immune modulation, limiting direct extrapolation to in vivo conditions [32]. Together, these model-specific characteristics underline the importance of interpreting propolis’ mechanistic effects within the biological context of each system and highlight the need for integrated approaches that bridge in vitro, animal and ultimately human studies.

### Mechanistic pathways: adipogenesis, Browning and lipid regulation

The anti-obesity potential of propolis is mediated through its modulation of adipogenesis, white adipose tissue (WAT) browning and lipid metabolism three interconnected metabolic processes. Propolis polyphenols, such as CAPE, chrysin, quercetin and artepillin C, downregulate key adipogenic transcription factors like PPARγ, C/EBPα and FAS, thereby inhibiting adipocyte differentiation and lipid accumulation. These anti-adipogenic effects are comparable to those seen with other natural polyphenols such as resveratrol and curcumin, which also inhibit adipocyte differentiation and promote thermogenesis through downregulation of PPARγ and related pathways [33–35]. This suggests that polyphenol-rich compounds, including propolis, may exert converging metabolic effects despite differing sources and structures. However, propolis composition and dosage appear to influence outcomes, as highlighted by contradictory findings from different propolis extracts [25,36,37].

Multiple studies confirm that propolis exerts anti-adipogenic effects by suppressing key transcription factors involved in fat cell differentiation (Table 2). For instance, Shin et al. (2014) demonstrated that CAPE-enriched propolis supplementation in HFD-induced mice significantly downregulated markers such as C/EBPα, PPARγ and FAS, resulting in reduced fat mass and body weight [23]. Likewise, Ichi et al. (2009) found decreased PPARγ expression and lower white fat tissue weight in Wistar rats treated with propolis. These findings are consistent with the effects of other polyphenol-rich compounds like resveratrol and curcumin, which inhibit adipogenesis through similar molecular targets [33,38,39].

However, the effects of propolis are not entirely consistent across studies. Lio et al. (2010) reported that red propolis extract enhanced adipocyte differentiation in 3T3-L1 cells, suggesting that the efficacy of propolis may vary depending on its chemical composition and dosage [25]. This variability is likely due to the diverse polyphenol content in propolis, such as artepillin C, quercetin, catechin and chlorogenic acid, which can influence metabolic outcomes by activating AMPK signaling, stimulating mitochondrial biogenesis and promoting sympathetic activity [17]. Furthermore, compounds like kaempferol and chrysin regulate the PXR/CYP3A4 pathway and may serve as markers for quality control in propolis-based interventions [40]. Dose–response studies also suggest a nonlinear relationship, with significant BMI and waist circumference reduction observed up to 8 weeks of supplementation before effects plateau [41]. These findings underscore the urgent need for standardization of propolis formulations to optimize its anti-obesity efficacy.

In parallel, propolis promotes the browning of WAT, converting energy-storing white adipocytes into thermogenic beige cells [42]. This process involves the upregulation of mitochondrial biogenesis and thermogenic markers such as UCP1, PRDM16 and PGC-1α. Artepilin C, for instance, enhances thermogenesis via creatine-dependent pathways, while chrysin induces both UCP1-dependent and independent mechanisms [17]. These findings underscore propolis’s potential to enhance energy expenditure and reduce adiposity through thermogenic reprogramming. In general, propolis has a good safety profile with fewer side effects when compared to pharmacological therapies [43]. Whether short-term or long-term use, propolis is known to have positive effects on metabolic improvement in obese samples. In a 30-day study, mice fed a high-fat diet and induced with propolis significantly improved lipid profiles, reduced triglyceride and cholesterol levels and showed antioxidant activity [44].

Several studies have demonstrated that propolis promotes the browning of WAT by upregulating thermogenic genes such as *UCP1*, *PRDM16* and *PGC-1α*, leading to increased energy expenditure and improved metabolic outcomes (Table 2). For example, Nishikawa et al. (2016) showed that propolis supplementation (5–100 mg/kg) elevated *UCP1* expression in both brown and white fat, while Cho et al. (2016) reported that New Zealand propolis suppressed ERK activity, a key regulator of thermogenesis [22,28]. Variations in propolis efficacy across regions are likely due to differences in bioactive components such as artepillin C (ArtC), which activates *PPARγ* and stabilizes *PRDM16*, facilitating the development of beige adipocytes [28,45].

Polyphenols in propolis also stimulate AMPK signalling and mitochondrial biogenesis, both essential for the thermogenic process [17]. Notably, ArtC enhances beige fat thermogenesis via creatine metabolism pathways, independent of β3-adrenergic signalling [28]. Similarly, chrysin, a flavonoid in propolis, promotes both *UCP1*-dependent and -independent thermogenesis by engaging ATP-consuming cycles involving creatine and calcium [15]. Collectively, these mechanisms support the role of propolis as a promising natural agent to stimulate WAT browning and combat obesity.

Moreover, Propolis influences lipid metabolism by downregulating key lipogenic genes such as *SREBP-1*, *FAS* and *ACC*, while simultaneously enhancing fatty acid oxidation [10,16]. Koya et al. (2009) demonstrated that propolis supplementation in Wistar rats reduced adiposity by suppressing *SREBP-1* and *FAS* expression [27]. Similarly, Cai et al. (2019) reported that ethanolic extract of propolis improved insulin sensitivity and boosted energy expenditure, reinforcing its role in lipid modulation [24]. Artepillin C (APC), a major bioactive compound in Brazilian green propolis, inhibits the *CREB/CRTC2* transcription complex, thereby downregulating *SREBP* and reducing hepatic and serum lipid levels [46]. This upstream suppression also leads to reduced expression of *FAS* and *ACC*, enzymes critical for fatty acid synthesis and storage. Furthermore, propolis activates the *Nrf2* pathway, which enhances mitochondrial biogenesis and lipid catabolism, providing an additional mechanism for its anti-obesity effects [47].

**Figure 2.**
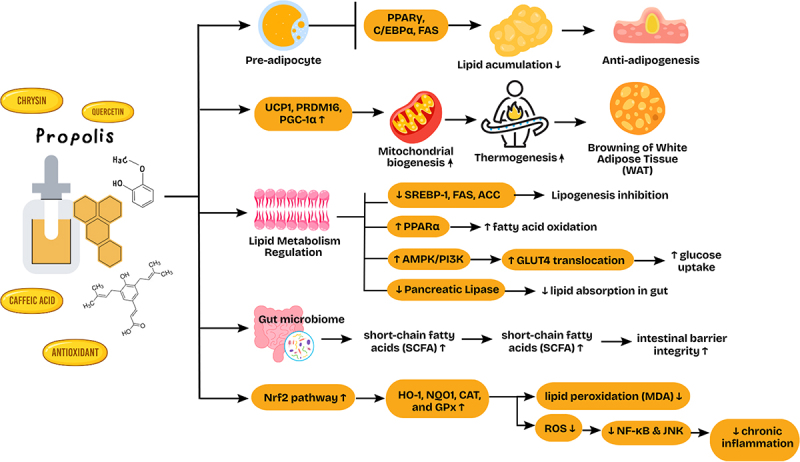
Proposed mechanisms of propolis in adipogenesis, lipid metabolism, and white adipose tissue Browning.

Beyond adipogenesis and browning, another important mechanism by which flavonoids in propolis may contribute to anti-obesity effects is through inhibition of pancreatic lipase, the key enzyme responsible for dietary fat digestion. Pancreatic lipase inhibition reduces triglyceride hydrolysis and subsequent fat absorption, thereby lowering postprandial lipid levels, and many flavonoids such as quercetin, chrysin and catechins have been identified as natural inhibitors of this enzyme [38,39,48]. This positions pancreatic lipase as a validated anti-obesity target that propolis-derived compounds may modulate. In addition, PPARα has also been validated as a central regulator of lipid catabolism and energy balance, and several flavonoids have been shown to activate PPARα, thereby enhancing fatty acid β-oxidation, reducing hepatic steatosis and improving lipid profiles [8,20]. Such mechanisms complement the previously discussed actions on PPARγ and AMPK, suggesting that propolis targets multiple nuclear receptors in its metabolic effects. Importantly, the bioactivity of propolis depends on its chemical composition; for example, caffeic acid phenethyl ester (CAPE) typically constitutes 1–2% of poplar-type propolis, whereas total flavonoid content may range from 5% to 15% depending on botanical origin [12,34,35]. These compositional data provide essential context for mechanistic interpretation, and the representative concentrations of CAPE, chrysin and quercetin are summarized in Figure 2. Together, these pathways highlight that the anti-obesity effects of propolis extend beyond adipogenesis and browning to include inhibition of lipid absorption and activation of fatty acid oxidation, thereby broadening its therapeutic potential.

### Anti-inflammatory and antioxidant mechanisms in obesity management

Obesity-induced chronic low-grade inflammation contributes to metabolic dysfunction. Resinous substances found in propolis provide anti-inflammatory and antioxidant effects that are relevant to obesity management. A study conducted by Cai et al. (2019) [24] revealed that EEP supplementation decreased inflammatory effects in obesity-induced mice. Also supported by Koya et al. (2009) who found that the content in Brazilian propolis can reduce inflammatory cytokines and improve metabolic health [27].

Propolis, especially its active component CAPE, has been shown to inhibit the NF-κB pathway to increase the expression of pro-inflammatory cytokines (TNF-α and IL-6) [49]. CAPE inhibits NF-κB by preventing the degradation of IκB, an inhibitor of NF-κB, thereby blocking the translocation of NF-κB into the nucleus where it should drive transcription of inflammatory genes [50]. CAPE, in particular, is known to paradoxically activate JNK in some contexts, which might contribute to its pro-apoptotic actions, but in general, it inhibits JNK activation in inflammatory situations [50]. The JNK pathway is another important signalling pathway involved in inflammation. Propolis and its components have been shown to inhibit JNK phosphorylation and activation, thereby reducing inflammatory responses [51,52]. CAPE, the main bioactive compound in propolis, has shown significant pharmacological activity against obesity-related conditions such as non-alcoholic fatty liver disease (NAFLD). CAPE inhibits bacterial bile salt hydrolase (BSH) activity in the gut, leading to increased levels of tauro-β-muricholic acid, which suppresses intestinal farnesoid X receptor (FXR) signalling. This suppression reduces ceramide levels, thereby reducing fat production and improving lipid metabolism [53].

Propolis activates the Nrf2 pathway, which increases the expression of antioxidant enzymes such as HO-1 and NQO1, thereby reducing oxidative damage [54]. Studies have shown that propolis reduces oxidative stress markers, such as ROS and lipid peroxidation, in various tissues, which is beneficial in managing obesity-related complications [55]. Propolis has been shown to significantly increase CAT and GPx activities in various studies. El-Haskoury et al.’s study revealed that in rats exposed to oxidative stress induced by carbon tetrachloride (CCl₄), propolis administration resulted in a significant increase in CAT and GPx activities, thereby reducing oxidative damage to liver and kidney tissues [56]. Propolis has been shown to reduce oxidative stress by reducing malondialdehyde (MDA) levels, a marker of lipid peroxidation, and enhancing the antioxidant defence system. Studies conducted on rats treated with nitric oxide synthase inhibitors, revealed propolis administration significantly decreased MDA levels and increased CAT activity in liver tissue, indicating a protective effect against oxidative stress [57].

### Other metabolic mechanisms of propolis in obesity management

Beyond its direct effects on adipogenesis, lipid metabolism and energy expenditure, propolis also modulates several metabolic disturbances commonly associated with obesity, namely hyperglycaemia, insulin resistance, hyperlipidaemia, oxidative stress and gut dysbiosis. These pleiotropic actions enhance its therapeutic promise in comprehensive obesity management.

#### Glycaemic control and insulin sensitivity

Propolis contains polyphenolic compounds, such as kaemferide, artepillin C and coumaric acid, that have been shown to improve glucose homoeostasis through multiple pathways. These compounds promote the translocation of glucose transporter 4 (GLUT4) to the cell membrane in skeletal muscle, enhancing glucose uptake via phosphorylation of the PI3K and AMPK pathways [58]. Additionally, artepillin C suppresses hepatic gluconeogenesis by inhibiting the CREB/CRTC2 transcription complex, thereby reducing fasting blood glucose and improving insulin sensitivity [46]. Propolis also downregulates glucose-6-phosphatase (G6Pase) expression by modulating GSK3α/β phosphorylation [59].

Under conditions of hyperglycaemia-induced oxidative stress, propolis exerts insulin-sensitizing effects through its antioxidant activity. Enzymes like catalase (CAT) and superoxide dismutase (SOD) are upregulated, reducing oxidative damage that impairs insulin signalling [60]. Moreover, compounds such as tectochrysin (TEC) mimic insulin action by activating insulin receptors, further enhancing glucose uptake in adipose and muscle tissue [61]. Inhibition of α-glucosidase activity by certain constituents, such as 3,4,5-tri-caffeoylquinic acid, also contributes to lowering postprandial blood glucose [62].

#### Lipid regulation and cholesterol modulation

In the context of obesity-related dyslipidemia, propolis significantly reduces total cholesterol and low-density lipoprotein-cholesterol levels [63,64]. These effects are mediated by enhanced hepatic expression of lipid transporters, including ABCA1, ABCG9, LDLR and SR-B1 [64], which facilitate reverse cholesterol transport. Furthermore, propolis inhibits HMG-CoA reductase activity, similar to the action of statins, potentially reducing endogenous cholesterol synthesis [65]. Its lipase-stimulating effect may also promote lipid clearance and elevate high-density lipoprotein cholesterol levels [44].

#### Gut microbiota and intestinal barrier function

Emerging evidence suggests that propolis exerts prebiotic-like effects, modulating gut microbiota composition and promoting the production of short-chain fatty acids (SCFAs) such as acetate, propionate and butyrate [52,66–68]. These metabolites play key roles in energy metabolism, insulin sensitivity and appetite regulation. Additionally, propolis strengthens intestinal barrier integrity by enhancing tight junction protein expression and reducing endotoxemia, thereby attenuating systemic inflammation [69,70]. This microbiota-mediated pathway may also influence polyphenol bioavailability and overall metabolic outcomes [10,71–73]. Collectively, these mechanisms demonstrate that propolis affects multiple aspects of metabolic syndrome. It not only targets obesity at the level of adipose tissue function and energy balance but also addresses comorbid metabolic disturbances such as insulin resistance, dyslipidemia and gut barrier dysfunction. These multi-target effects support the potential of propolis as a complementary approach in obesity therapy, although further standardized clinical studies are needed to confirm its translational relevance. The proposed mechanism of propolis, as summarized in Figure 2, highlights its capacity to regulate adipogenesis, lipid metabolism and white adipose tissue browning, which may play a pivotal role in mediating systemic metabolic improvements (Figure 2).

Taken together, current preclinical evidence highlights the therapeutic promise of propolis in modulating adipogenesis, enhancing thermogenesis and improving lipid metabolism in obesity models. However, its clinical translation remains hindered by significant challenges, including compositional variability across geographic sources, inconsistent dosing protocols, limited understanding of bioavailability and a paucity of human trials. Although propolis appears safe within studied doses and durations, the absence of long-term toxicity and efficacy data in humans precludes its immediate adoption in obesity management guidelines. Translational considerations doses used in rodents (e.g. 5–50 mg/kg) cannot be directly compared to human mg/kg doses without appropriate allometric scaling and pharmacokinetic data. Simple mg/kg ratios may overestimate human-equivalent exposure; established methods for interspecies dose conversion (e.g. allometric scaling) should be applied when estimating a human-equivalent dose [74]. Moreover, variability in propolis composition between geographical origins further complicates dose translation. Consequently, clinical trials with standardized preparations and pharmacokinetic assessments are required before recommending clinical dosing.

## Conclusion

This systematic review highlights the therapeutic potential of propolis and its bioactive components in obesity management through multiple mechanisms, including the inhibition of adipogenesis, promotion of white adipose tissue browning, modulation of lipid metabolism and reduction of inflammation. These effects are primarily supported by preclinical evidence, suggesting that propolis may enhance metabolic health by targeting key molecular pathways such as AMPK, PPARγ and UCP1. However, it is important to recognize that most findings are derived from animal models or in vitro studies, with limited data from well-designed clinical trials. Therefore, while promising, these results should be interpreted as exploratory and hypothesis-generating rather than conclusive. Future studies must focus on clinical validation, optimal dosage determination and safety profiling in human populations.

A major challenge that remains is the standardization of propolis, given its significant chemical variability depending on geographical and botanical sources. This heterogeneity may affect reproducibility and complicate translational application. Furthermore, this review is limited by the small number of eligible studies, potential publication bias and the lack of quantitative meta-analysis due to heterogeneity in study design, propolis composition and outcome measures. In conclusion, propolis shows encouraging anti-obesity potential, but critical gaps in evidence must be addressed through rigorous, standardized and translational research to support its use as a complementary therapeutic strategy in clinical settings.

## Supplementary Material

527269_Supplementary File_Tables S1_Search Strategy.docx

## Data Availability

The data that support the findings of this study are available from the corresponding author upon reasonable request.
