# Supplementary material for: Molecular mechanisms of propolis in adipogenesis, lipid metabolism and white adipose tissue Browning: a systematic review of preclinical studies
Source: Adipocyte. 2025 Oct 18;14(1):2576894. doi: 10.1080/21623945.2025.2576894 (PMC12536616; doi:10.1080/21623945.2025.2576894)
Supplement: 527269_Supplementary File_Tables S1_Search Strategy.docx [file KADI_A_2576894_SM5124.docx]

**SUPPLEMENTARY FILES**

**Table S1**. Search Strategy

| No | Database/  Search Engine | Searching Strategy | Result |
| --- | --- | --- | --- |
| 1. | PubMed | (propolis or lee glue) AND (obesity OR lipid metabolism OR tissue browning OR thermogenesis OR white adipose tissue OR weight loss) | 292 |
| 2. | Scopus | TITLE-ABS-KEY (propolis OR "bee glue") AND ( obesity OR lipid AND metabolism OR tissue AND browning OR thermogenesis OR white AND adipose AND tissue OR weight AND loss ) | 388 |
| 3 | Google Scholar | propolis OR "Bee products" AND obesity AND adipogenesis OR "Lipid Metabolism" OR "White Adipose Tissue Browning" OR thermogenesis OR "Weight loss" | 3.220 |
| TOTAL | | | 3900 |
